# Efficacy and Safety of Endoscopic Strictureplasty and Stricturotomy for Crohn’s Disease-Associated Strictures: A Systematic Review and Current Perspective

**DOI:** 10.3390/diseases14040121

**Published:** 2026-03-27

**Authors:** Elisa Abreu, Rolando Pinho, Fernando Magro, Maria Manuela Estevinho

**Affiliations:** 1 Faculty of Medicine, University of Porto, 4200-319 Porto, Portugal; eclsa@hotmail.com; 2 Department of Gastroenterology, Unidade Local de Saúde Gaia Espinho (ULSGE), 4434-502 Vila Nova de Gaia, Portugal; 3 Department of Medical Sciences, University of Aveiro, 3810-193 Aveiro, Portugal; 4 CINTESIS@RISE, Department of Community Medicine, Information and Health Decision Sciences (MEDCIDS), Faculty of Medicine of the University of Porto (FMUP), 4200-450 Porto, Portugal; 5 Department of Gastroenterology, Unidade Local de Saúde São João (ULSSJ), 4200-319 Porto, Portugal; 6 Unit of Pharmacology and Therapeutics, Department of Biomedicine, Faculty of Medicine, University of Porto, 4200-319 Porto, Portugal

**Keywords:** Crohn’s disease, endoscopic strictureplasty, endoscopic stricturotomy, fibrostenotic disease, intestinal strictures, therapeutic endoscopy

## Abstract

Objectives: Strictures are a major complication of Crohn’s disease (CD) affecting up to 20% of patients at diagnosis. Endoscopic balloon dilation (EBD) is the first-line endoscopic approach; however, it entails complications and a need for reintervention. Endoscopic stricturotomy (ESt) and stricturoplasty (ESTx) are promising alternatives. This review aims to provide an up-to-date and comprehensive assessment of their efficacy and safety in CD-associated strictures. Methods: A literature search was performed until August 2025. Primary outcomes were clinical and technical success. Secondary outcomes included adverse events, additional endoscopic or surgical treatments, medication escalation, emergency department visits and hospitalization following intervention. A minimum of four studies were required for meta-analysis, and pooled estimates were calculated using random-effects meta-analysis. Study quality was assessed using CASP checklist. Results: Fifteen studies including 1050 IBD patients (470 CD) were included. Strictures were short (0.9–2.4 cm) and some had prior EBD (7.8–57.1%) or surgery (3.6–91%). Technical success of ESt ranged from 88% to 100% and clinical success from 50% to 96%. The bleeding rate was up to 11.8%, but perforation rate was mostly <2%. The need for additional intervention, endoscopic (18.2–66.6%) or surgical (0–18.2%), varied considerably. Additionally, ESTx’s technical success ranged from 91.7% to 100% whereas clinical success ranged from 71.4% to 91%, with bleeding ranging from 5.2% to 8.8% and perforation from 0% to 3.4%. Similarly, the need for additional endoscopic procedures (7.1–57.1%) and surgery (9.5–25%) varied considerably. Conclusions: ESt and ESTx are safe and effective for managing CD-related strictures, particularly when short, straight, accessible, fibrotic, anastomotic, or refractory to EBD.

## 1. Introduction

Crohn’s disease (CD) is a chronic inflammatory disorder of the gastrointestinal tract. One of the most significant and challenging complications of CD is the development of intestinal strictures that can lead to higher risk for hospitalization, steroid dependency, repeated endoscopy and surgery [1].

Strictures are particularly prevalent in CD, with up to approximately 20% of patients presenting with stricturing phenotype at diagnosis [2,3]. In a recent European population-based inception cohort of 488 patients, approximately 10% of patients with non-complicated CD progressed to stricturing disease at the end of the 5-year follow-up [3]. Intestinal strictures can be categorized into inflammatory, fibrostenotic, and mixed types by endoscopy, histology, and cross-sectional imaging [4]. Whereas lesions with a larger inflammatory component are more amenable to medical therapy, this therapy alone is often insufficient to resolve established fibrotic strictures [5]. Therefore, endoscopic and surgical interventions play a crucial role in the effective management of these lesions.

Endoscopic balloon dilation (EBD) remains the first-line endoscopic approach for the treatment of CD-associated strictures. In a systematic review and meta-analysis, it was associated with a pooled technical success rate of 94.9% and symptomatic relief of 82.3% [6]. However, EBD is associated with non-negligible risks, including major complications in 5.3% of patients, comprising bleeding, perforation or dilation-related surgery, and a significant rate of symptom recurrence (48.3% of patients) and need for reintervention, with 38.8% undergoing repeat dilation and 27.4% requiring surgery during a mean follow-up of 20.5 months [6]. While surgical resection or strictureplasty remains the definitive option for refractory or complex strictures, its associated morbidity, postoperative recurrence (up to 25%), and cumulative bowel loss highlight the need for effective, bowel-sparing endoscopic alternatives [7,8].

In light of these limitations, endoscopic stricturotomy (ESt) and stricturoplasty (ESTx) have emerged as promising alternative approaches. ESt consists of targeted electrosurgical incision of the stricture until adequate endoscope passage is achieved, whereas ESTx involves the placement of endoclips at the incision site after stricture incision, with the aim of enhancing both short-term and long-term luminal patency [9].

Despite its promising outcomes, the evidence supporting the routine use of Est and ESTx in IBD remains limited, especially in comparison to more established modalities, like EBD. This lack of consolidated data represents a significant barrier to their wider clinical adoption. To address this unmet need, the present systematic review aims to provide the most comprehensive and updated assessment to date of the efficacy and safety of EST and ESTx in CD-associated strictures. By synthesizing the available evidence, we seek to clarify their therapeutic value and support more informed and evidence-based clinical decision-making.

## 2. Materials and Methods

The systematic review with metanalysis was conducted in accordance with the Preferred Reporting Items for Systematic Reviews and Meta-Analyses (PRISMA) guidelines and was registered in PROPERO (ID CRD420251066872).

### 2.1. Literature Search

A comprehensive search of the literature was performed using PubMed, Web of Science, and Scopus to identify studies evaluating endoscopic management of CD-associated strictures. The search included all studies published up to 31 August 2025. The following terms were used for the search: (“endoscopic stricturotomy” OR “endoscopic strictureplasty” OR “endoscopic stricturoplasty” OR “endoscopic strictur*” OR “endoscopic electroincision” OR “needle knife” OR “endoscopic incision” OR “ESt” OR “ESTx”) AND (“IBD” OR “inflammatory bowel diseas*” OR “Crohn*” OR “ulcerative colitis”)—Table A1. In addition, a manual search of the abstract book from the European Crohn’s and Colitis Organisation (ECCO) Congress 2026 was performed to identify potentially relevant studies not captured in the electronic database search and to ensure inclusion of the most up-to-date evidence. Two investigators (EA and MME) independently performed the literature search, screened titles and abstracts, and reviewed full texts for eligibility. The process was managed using Rayyan software. Any disagreements between reviewers were resolved through discussion and, when necessary, by consultation with a third investigator (FM).

### 2.2. Study Selection

Studies were considered eligible if they met the following inclusion criteria: (i) included patients with CD who underwent endoscopic therapy; (ii) the endoscopic intervention involved endoscopic stricturotomy and/or strictureplasty; and (iii) technical and/or clinical success were reported. Both interventional and observational studies were eligible for inclusion. Studies were excluded if they were non-original works (such as reviews, book chapters, commentaries, letters, editorials, press articles, or clinical guidelines), in vitro or animal studies, or case reports and case series including less than 10 patients.

### 2.3. Data Extraction

Two reviewers independently extracted data from the included studies using a standardized data collection form. Extracted data included the following: study identification (authors, year), study design and recruitment period; population characteristics (number of patients, disease phenotype when available), prior treatments (in endoscopic or surgical interventions, use of immunosuppressive medications), and stricture features (number, location and morphology when available); details of the endoscopic procedure (stricturotomy or strictureplasty), including tools or accessories used (e.g., endoscopic knives, clips); duration of follow-up; outcome definitions and measures of technical success, clinical success, and safety and reported rates of outcome achievement; and adverse events (e.g., bleeding, perforation, need for additional endoscopic procedures, surgery, or hospitalization), including numerators and denominators for outcome events. Any discrepancies in data extraction were resolved through discussion or consultation with a third reviewer.

### 2.4. Outcomes of Interest

The primary outcomes were technical success (generally defined as successful passage of the endoscope through the treated stricture immediately after the procedure) and clinical success (improvement in patient-reported symptoms following endoscopic intervention). The secondary outcomes included the following: incidence of adverse events, requirement for subsequent surgical intervention for stricture management, need for repeat endoscopic procedures, escalation of medical therapy following endoscopic treatment, and disease-related emergency department visits or hospitalizations.

### 2.5. Study Quality Assessment

The methodological quality of included studies was evaluated independently by two reviewers using the Critical Appraisal Skills Programme (CASP) checklist [10]. Each study was assessed across the checklist items and rated as yes, no, or can’t tell.

### 2.6. Statistical Analysis

When at least four studies reported data for a given outcome, a meta-analysis of proportions was performed. Pooled estimates and corresponding 95% confidence intervals (CIs) were calculated using a random-effects model, accounting for between-study heterogeneity. Proportions were logit-transformed prior to pooling. Statistical heterogeneity was assessed using the I^2^ statistic. Subgroup analyses were performed according to the type of publication (abstract versus full-text) and the endoscopic technique used in each study (ESt, ESt + ESTx, and ESt + EBD). Analyses were conducted in R (R Foundation for Statistical Computing, Vienna, Austria) using the meta, dplyr, and readxl packages.

### 2.7. Artificial Intelligence Use Statement

Artificial intelligence (AI) tools were used during the preparation of this article to assist in the generation of illustrative content. Specifically, selected components of Figure 2D–F were generated using the tool Nano Banana Pro, based on transformations of human images into conceptual representations. These images were created to support the visualization of intestinal stricture and its endoscopic treatment and do not represent real patient data. The authors critically reviewed and edited all AI-generated material to ensure scientific accuracy and appropriateness. 

## 3. Results

### 3.1. Characteristics of the Included Studies

**Figure A1:**
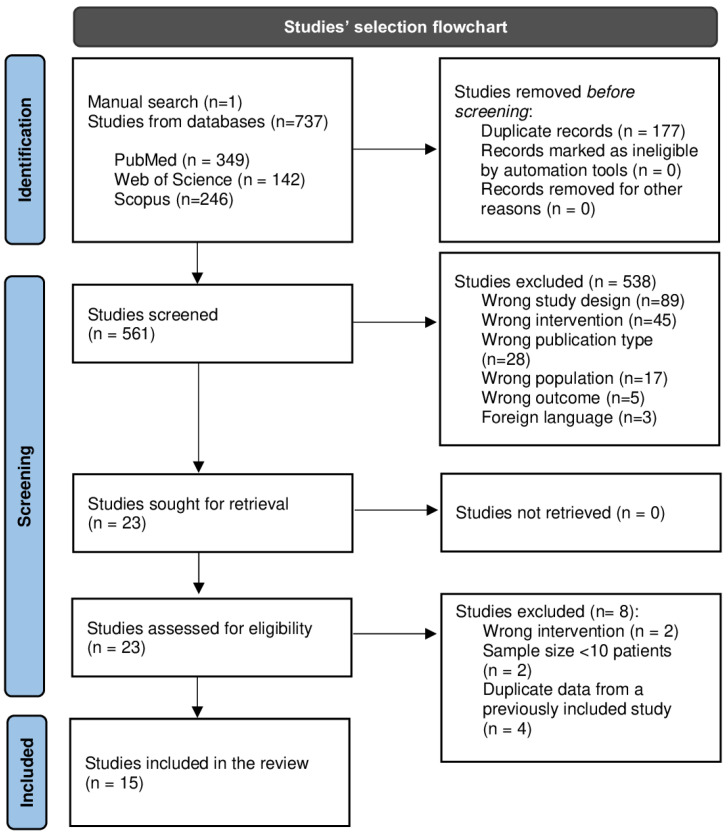
Study selection flowchart, following PRISMA 2020 guidelines [36].

The database search across PubMed, Web of Science, and Scopus identified 737 records. An additional record was identified through a manual search of the abstract book from the ECCO Congress 2026. After excluding 177 duplicates, 561 studies proceeded to title and abstract screening. From this, 538 were excluded, mostly because of wrong study design (reviews, case reports or case series with less than 10 patients), wrong intervention and wrong publication type (commentaries, letters or clinical guidelines). Full-text review was performed for 23 articles, resulting in the inclusion of 15 studies [11,12,13,14,15,16,17,18,19,20,21,22,23,24,25]. Of the eight studies excluded, two were excluded due to wrong intervention (had only performed EBD), two because of small sample size (<10 patients) and the other four due to duplicate data from other already included studies (Figure A1). Table 1 summarizes the study design, patient characteristics, and detailed stricture features, as well as the endoscopic procedures and primary outcomes of the included studies. The majority of included studies were retrospective cohort studies (n = 14), with a single randomized controlled trial [25]. Most studies originated from the USA [11,12,13,14,15,16,17,20,21,22,23], some from China [14,19,24], one from Czech Republic [18] and one from India [25]. Fourteen studies [11,12,14,15,16,17,18,19,20,21,22,23,24,25] enrolled a total of 1050 patients; one did not specify the number of enrolled patients and reported only the number of stricturing lesions. Across thirteen of the included studies [11,12,14,15,16,17,18,19,20,21,22,24,25] the number of patients with CD was specified, totalling 470 individuals. The included patients were mainly patients with Crohn’s disease, mostly located in the terminal ileum (L1) with stricturing phenotype (B2). Many had complicated or refractory disease, with prior exposure to corticosteroids [14,15,17,19,20,21,22,23,25] (from 3.6% [19] to 34.3% [14]), immunomodulators [14,15,17,23] (from 5.1% [23] to 38.5% [15]), and biologic therapies [11,12,14,15,16,17,19,20,22,23,25] (from 14.3% [19] to 76.5% [20]), and a substantial proportion had undergone previous EBD [11,12,14,16,17,18,22,25] (from 7.8% [25] to 57.1% [12]) or surgery [11,14,16,19,20,24] (from 3.6% [19] to 91% [16]). Patients’ characteristics are delineated in detail in Table A2.

**Table 1:** Characteristics of the included studies.

Study ID	Study Design	Study Period	Follow Up	Patients		Strictures’ Characteristics					Endoscopic Procedures	Primary Outcomes
				N.	Symptomatic	N.	Type	Length	Non- Traversable	Location		
Lan, 2017, USA [11]	Retrospective cohort	2008–2016	Median: 0.9 year (IQR 0.3–1.8)	n = 85 (85 with IBD, 35 with CD)	NR	127	Primary: 59 (69.4%) patients Secondary: 33 (38.8%) patients	Median: 1.5 cm (IQR 1.0–2.0)	54/127 (42.5%)	Ileocecal valve: 8 (6.3%); pouch inlet: 22 (17.3%); pouch afferent limb: 25 (19.7%); middle of the pouch body: 3 (2.4%); ileal pouch anastomosis: 24 (18.9%); nipple valve of the Kock pouch: 2 (1.6%); ileocolic anastomosis: 22 (17.3%); ileorectal anastomosis: 2 (1.6%); colocolonic anastomosis: 1 (0.8%); distal ileum: 12 (9.4%); anal canal: 5 (3.9%); colon: 1 (0.8%)	ESt + EBD (14 strictures) (n = 272)	Technical success Surgery-free survival
Lan, 2018, USA [12]	Retrospective cohort	2009–2016	Median: 0.8 year (IQR 0.1–1.6)	n = 185 (21 in the ESt arm: 21 with IBD, 21 with CD)	15/21 (71.4%)	45	Anastomotic	Median: 1.5 cm (IQR 1.0–2.4)	12/21 (57.1%)	Ileocolonic: 18 (85.7%); ileorectal: 2 (9.5%); colocolonic: 1 (4.8%)	ESt + ESTx (n = 45)	Surgery-free survival Post-procedural complication
Lan, 2019, USA [13]	Retrospective cohort	2008–2016	NR	NR	NR	84	Primary: 47 (56%) Anastomotic: 37 (44%)	NR	NR	NR	ESt (n = NR)	Surgery-free survival
Lan, Stocchi, 2019, USA, China [14]	Retrospective cohort	2010–2017	Median: 0.8 year (IQR 0.2–1.7)	n = 182 (35 in the ESt arm: 35 with IBD, 35 with CD)	30/35 (85.7%)	49	Anastomotic	Median: 2.0 cm (IQR 1.5–2.5)	21/31 (67.7%)	Ileocolonic: 35/35 (100%)	ESt (n = 49)	Surgery-free survival Post-procedural complication
Lan, 2020, USA [15]	Retrospective cohort	2001–2016	Median: 1.8 year (IQR 1.1–2.4)	n = 45 (13 in the ESt arm: 13 with IBD, 13 with CD)	9/11 (81.8%)	29	Primary	Mean ± SD: 2.4 ± 0.9 cm	13/13 (100%)	Distal ileum: 13/13 (100%) within 15 cm from the ileocecal valve and/or at the ileocecal valve	ESt (n = 29)	Surgery-free survival
Mohy-Ud-Din, 2020, USA [16]	Retrospective cohort	2018–2020	Mean ± SD: 0.4 ± 0.3 year	n = 11 (11 with IBD, 7 with CD)	11/11 (100%)	12	Primary: 1 (8.3%) Anastomotic: 11 (91.7%)	Mean ± SD: 1.3 ± 0.4 cm	11/12 (91.7%)	Ileo-colonic: 6 (50%); J-pouch: 2 (17%); anal canal: 2 (17%); rectal cuff: 1 (8%); terminal ileum: 1 (8%)	ESt + ESTx (n = NR)	Technical success
Zhang, 2020, USA [17]	Retrospective cohort	2009–2016	Median: 0.9 year (IQR 0.3–1.6)	n = 64 (49 with IBD, 25 with CD)	34/49 (82.9%)	NR	Anastomotic	Mean ± SD: 1.8 ± 0.8 cm	27/49 (55.1%)	IPA: 24 (48.9%); ileocolonic: 22 (44.9%); ileorectal: 2 (4.1%); colocolonic: 1 (2.0%)	ESt (n = 106)	Technical success Surgery-free survival
Lukas, 2022, Czech Republic [18]	Retrospective cohort study	2018–2021	Mean ± SD: 1.5 ± 0.8 year	n = 67 (67 with IBD, 60 with CD)	NR	92	Primary: 10 (10.9%) Anastomotic: 82 (89.1%)	NR	NR	Primary: anal canal 10 (10.9%) Anastomotic: ileocolonic: 59 (64.1%); colocolonic: 9 (9.8%); ileorectal: 3 (3.3%); IPA: 11 (12.0%)	ESt (n = NR)	Technical success Complications
Ning, 2023, China [19]	Multicentre retrospective cohort	2017–2023	Median: 1.4 year (IQR 0.8–2.0)	n = 28 (28 with IBD, 28 with CD)	28/28 (100%)	57	Fibrotic	Median: 1 cm (IQR 1.0–1.9)	57/57 (100%)	Jejunum: 11 (19.3%); ileum: 46 (80.7%)	ESt + ESTx (n = 58)	Technical success Short-term and long-term clinical efficacy
Kochhar, 2023, USA [20]	Retrospective cohort	N/A	Mean: 1.1 year	n = 149 (68 in the ESt arm: 68 with IBD, 68 with CD)	NR	NR	Primary: 44 (64.7%) patients Anastomotic: 24 (35.3%) patients	NR	NR	Small bowel: 29/68 (42.6%); colonic: 39/68 (57.4%)	ESt (n = NR)	Time to repeat endoscopic intervention
Khan, 2024, USA [21]	Retrospective cohort	2018–2023	NR	n = 48 (48 with IBD, 48 with CD)	NR	NR	Primary (NAS): 18 (37.5%) patients Anastomotic (AS): 30 (62.5%) patients	Mean ± SD: NAS: 1.58 ± 0.36 cm; AS: 0.9 ± 0.5 cm	NR	Colon is the most frequent stricture location in both: NAS: 27.7% AS: 33.3%	ESt + EBD (11 patients) (n = NR)	NR
Herman, 2024, USA [22]	Retrospective cohort	2020–2022	Mean ± SD: 1.1 ± 0.5 year	n = 24 (24 with IBD, 18 with CD)	NR	NR	NR	Mean ± SD: 2.4 ± 1.2 cm	24/24 (100%)	Anorectal: 17 (71%) Anopouch: 7 (29%)	ESt (n = NR)	Technical success
Chaudhary, 2024, USA [23]	Retrospective cohort	2018–2023	NR	n = 50 (39 in the ESt arm: 39 with IBD, NR with CD)	NR	NR	Primary: 13 (33.3%) patients Anastomotic: 24 (61.5%) patients	Mean ± SD: 1.2 ± 1 cm	NR	Ileocolonic: 5 (12.8%); ileorectal: 1 (2.5%); inlet and loop ileostomy: 1 (2.5%); anal: 3 (7.6%); cecum: 2 (5.1%); colonic: 14 (35.8%); ileum: 7 (17.9%); ileocecal anastomosis: 1 (2.5%); rectum: 3 (7.6%); recto-sigmoid colon: 2 (5.1%)	ESt (n = NR)	NR
Cui, 2025, China [24]	Retrospective cohort	2020–2024	NR	n = 11 (11 with IBD, 11 with CD)	NR	17	NR	NR	NR	Ileocecal valve: 7 (41.2%) Ileum: 5 (29.4%) Ascending colon: 1 (5.9%) Proximal ascending colon: 1 (5.9%) Duodenum: 2 (11.8%) Rectum: 1 (5.9%)	ESt + EBD (5 patients) (n = 17)	Immediate success Remission time Surgical treatment Re-endoscopic intervention Complications
Pal, 2026, India [25]	Randomized control study	2022–2025	Median: 1 year (IQR 0.25–3)	n = 101 (51 in the ESt arm; 51 with IBD, 51 with CD)	51/51 (100%)	NR	Primary: 43 (84.3%) Anastomotic: 8 (15.7%)	Median: 1.5 cm (IQR 0.5–3)	51/51 (100%)	Upper GI: 2 (3.9%) Ileal: 10 (19.6%) Ileocecal: 13 (25.5%) Ileal + ileocecal: 3 (5.9%) Colonic: 14 (27.5%) Anorectal: 9 (17.6%)	ESt	Clinical recurrence at 1 year

AS, anastomotic strictures; CD, Crohn’s disease; EBD, endoscopic balloon dilatation; ESTx, strictureplasty; ESt, stricturotomy; IBD, inflammatory bowel disease; IPA, ileal pouch-anal; NAS, non-anastomotic strictures; NR, not reported.

Six studies [15,18,19,20,22,25] reported follow-up periods equal to or longer than one year, while another five [11,12,14,16,17] reported follow-ups between a mean of 4.8 months and a median of 10.8 months. The remaining four studies [13,21,23,24] did not specify their follow-up duration. Nine studies [11,12,13,14,15,16,18,19,24] reported a total of 512 strictures, while the remaining six [17,20,21,22,23,25] did not specify. Stricture length, reported in ten studies [11,12,14,15,16,17,19,21,22,23,25], was generally short, with median lengths ranging from 1 to 2 cm and means lengths varying between 0.9 cm and 2.4 cm. Stricture location varied among the studies, with some reporting primary (n = 1), anastomotic strictures (n = 3), or a combination of both (n = 8) occurring at various locations. The endoscopic procedures performed across all studies were ESt alone [11,13,14,15,17,18,20,21,22,23,24,25] or ESt combined with ESTx [12,16,19]. However, in three studies [11,21,24] some patients underwent combined therapy with ESt and EBD. Specifically, in [11], 14 of 272 strictures were treated with ESt and EBD; in [21], EBD was performed concurrently in 11 of 48 patients; and in [24], 5 of 11 patients were treated.

### 3.2. Methodological Quality

All studies addressed a clearly focused issue: the safety and efficacy of ESt and/or ESTx. The majority were conducted in tertiary referral centres, although one study used a multicentre design [19]. Six [13,18,20,21,23,25] of the 15 included studies, were abstracts whose full article was not available/published. For these studies there was information lacking regarding inclusion and exclusion criteria, exposure and outcome measurement, as well as confounding factors and follow-up data (Table A3). Regarding the remaining, most clearly defined and described the intervention (ESt and/or ESTx), relying on medical records to assess exposure and outcomes. However, in five [11,12,14,16,17] the follow-up was short (less than 1 year). Finally, some recognized potential confounders were age at CD diagnosis, gender, body mass index, disease location and behaviour, length and degree of stricture, and previous procedures [11,12,14,19,25]. And in some studies, an analysis was conducted to evaluate which factors could be associated with need [11,12] or time to reintervention [18] and surgery-free survival [14,15,19]. One [14] used propensity matching to compare the outcomes. Additionally, [25] conducted a multivariate analyses to find predictors of clinical recurrence and reintervention. Four [15,16,17,22] studies did not consider confounding factors in analysis, and no study evaluated factors potentially associated with technical or clinical success.

### 3.3. Endoscopic Stricturotomy (ESt)

**Figure 1:**
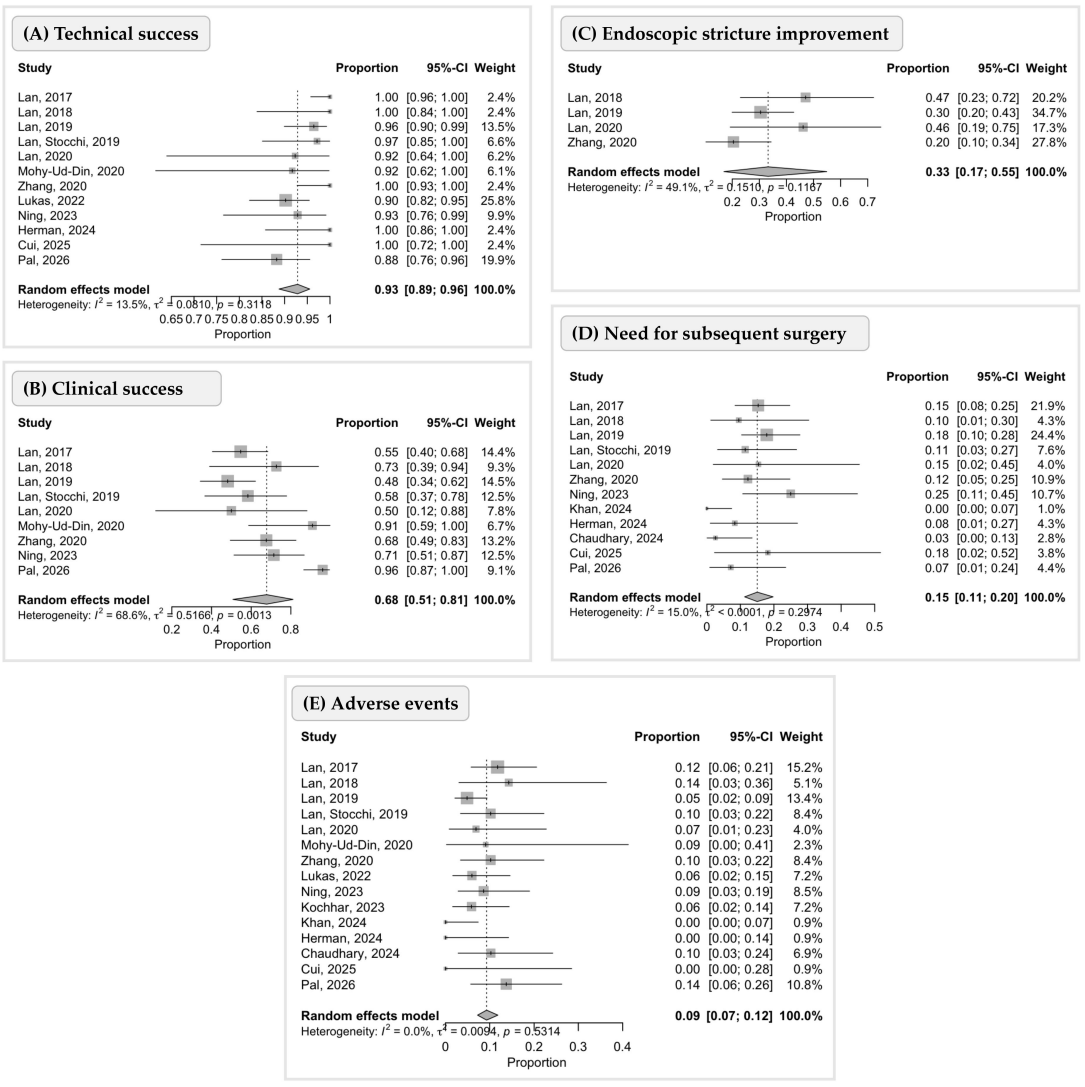
Forest plots showing pooled estimates of (**A**) technical success, (**B**) clinical success, (**C**) endoscopic stricture improvement, (**D**) need for subsequent surgery, and (**E**) adverse events following endoscopic stricturotomy for CD-associated strictures. Study weights are indicated by the size of the squares, and horizontal lines represent 95% confidence intervals [11,12,13,14,15,16,17,18,19,20,21,22,23,24,25].

Most studies (n = 7) [12,14,15,16,17,19,24] provided a clear description of the technique and materials used for stricturotomy. In general, the procedure was defined as follows: after careful endoscopic assessment of the stricture, an electrosurgical knife was used to perform, semicircumferential, horizontal, or full circumferential incisions along the stricture under direct visualization to expand the narrowed lumen. The material used for the procedure varied across studies. The most commonly used electrosurgical knives were the triple-lumen needle knife (Boston Scientific, Marlborough, MA) (n = 5) and the IT Knife 2 (Olympus Medical Systems, Tokyo, Japan) (n = 6), used with Endocut mode on an ERBE electrosurgical generator. The settings and other details are presented in Table 2. The primary efficacy outcomes reported were technical and clinical success. Technical success was defined as the immediate confirmation of luminal patency, demonstrated by successful passage of the endoscope through the treated stricture [11,12,14,15,16,17,18,19,22,24,25]. Clinical success was defined as the improvement in patient-reported symptoms following intervention [11,12,13,14,15,16,17,19,25]. Technical success was consistently high across most studies: five studies [11,15,17,22,24] reported a 100% technical success rate and four [13,14,18,25] reported rates ranging from 88% to 97%. In the pooled analysis, depicted in Figure 1, technical success was 93% (95% CI 89–96%), with low heterogeneity (I^2^ = 13.5%). However, clinical success was notably lower, ranging from 50% [15] to 96% [25]. The pooled clinical success rate was 68% (95% CI 51–81%), with substantial heterogeneity (I^2^ = 68.6%)—Figure 1. Unfortunately, some recent studies did not report either technical [20,21,23] or clinical [18,20,21,22,23,24] success (Table 3).

Endoscopic stricture improvement was reported in a limited number of studies and showed variable rates across individual reports. In pooled analysis, endoscopic stricture improvement was observed in 33% of cases (95% CI 17–55%), with moderate heterogeneity (I^2^ = 49.1%)—Figure 1.

The need for additional endoscopic procedures during follow-up varied considerably. The highest endoscopic reintervention rates were observed in [22] (with 66.6% of patients requiring additional ESt in a mean time of 5.3 ± 4.0 months) and [11] (44.9% requiring ESt, 22.8% EBD and 11% ESt + EBD in a mean time of 7.0 ± 6.8 months). Conversely, ref. [24] reported a lower endoscopic reintervention rate of 18.2%. Rates of subsequent surgical intervention ranged from 0% [21] to 18.2% [24], the pooled rate being 15% (95% CI 11–20%), with low heterogeneity (I^2^ = 15.0%). Ref. [24] described a remission duration of 10.1 ± 8.2 months (time elapsed after the initial ESt treatment without the need for further endoscopic or surgical treatment).

Regarding safety outcomes of ESt, bleeding was the most reported immediate adverse event. The highest bleeding rate was 11.8% [25] while several studies reported no bleeding events [22,24]. Perforation rates were generally low, with most studies [11,14,17,20,22,24,25] reporting rates lower than 2%, ranging from 0% in multiple studies [17,20,22,24] to 15.4% [15]. In pooled analysis, adverse events were observed in 9% of cases (95% CI 7–12%), with no significant heterogeneity (I^2^ = 0.0%)—Figure 1. Stricture-related emergency department visits, hospitalizations and medical therapy escalation were mostly not reported; only five studies [11,14,15,17,25] provided data on these outcomes (Table 4).

Subgroup analyses stratified by publication type (abstract versus full-text) and endoscopic technique (ESt, ESt + ESTx, ESt + EBD) demonstrated comparable pooled estimates across groups. No statistically significant differences were observed between subgroups for any of the assessed outcomes (*p* > 0.05 for all outcomes) (Table A4).

### 3.4. Endoscopic Strictureplasty (ESTx)

Three studies [12,16,19] reported on the use of an endoscopic strictureplasty technique. This procedure consists of incision of the stricture followed by clip placement at the incision site to maintain luminal patency and reduce the risk of delayed bleeding (Table 2). Two studies [12,16] described that this procedure was applied routinely, whereas one study [19] stated that it was performed at the “discretion of the endoscopist for hemostasis”, suggesting that some patients were subjected solely to stricturotomy and others to strictureplasty. Therefore, in this report [19] it is not possible to determine whether the reported outcomes refer exclusively to strictureplasty or stricturotomy. Despite this, technical success was high across all three studies. Ref. [12] reported a technical success rate of 100%, while [16,19] reported rates of 92.9% and 91.7%, respectively. Clinical success varied between studies. Symptomatic improvement was reported in 72.7% of patients in [12] and in 71.4% in [19], whereas [16] reported a higher clinical success rate of 91% (Table 3).

Regarding safety outcomes of ESTx, the bleeding and perforation rates were similar between studies, with bleeding ranging from 5.2% [19] to 8.8% [12] per procedure and perforation from 0% [12,16] to 3.4% [19] per procedure. Overall adverse event rate was only reported by [19] (8.6%). The need for additional endoscopic procedures during follow-up varied considerably, with [12] reporting the highest rate (57.1%) and [19] the lowest (7.1%). On the other hand, [19] reported the highest rate of subsequent surgical intervention (25%) while [12] reported 9.5%. Stricture-related emergency department visits (9.5%), hospitalizations (4.8%) and medical therapy escalation (14.3%) were mostly reported by [12] (Table 4).

### 3.5. Additional Evaluated Outcomes

Finally, several studies reported additional outcomes. Four [12,13,15,17] assessed endoscopic stricture improvement, defined by [17] as a shorter or more patent stricture in the immediate follow-up endoscopy, with documented rates ranging from 20% [17] to 47% [12] (Table 3). Two studies evaluated clinical recurrence, defined by [25] as reoccurrence of initial symptoms or >1-point increase in CD obstruction score. Ref. [16] reported no clinical or endoscopic recurrence during a follow-up of 144 ± 105 days, while [25] reported a 24.5% rate at 1 year. Ten [11,12,13,14,15,17,18,19,22] used Kaplan–Meier analyses to assess the probability of reintervention. Two [18,19] assessed the cumulative reintervention-free rate (endoscopic or surgery reintervention): 59.7% (95% CI 45.4–74.5%) [18] or 63.7% (95% CI, 47–86.3%) [19] at 12 months and 51.2% (95% CI 37.9–66.0%) [18] at 18 months. On the other hand, [22] reported a 33% cumulative endoscopic reintervention-free rate during follow-up of 1.1 ± 0.5 year. And three studies [11,19,22] estimated the surgery-free rate: 3-year surgery-free survival rate of 62.0% [11], a 1-year rate of 74.8% [19], and a cumulative surgery-free survival of 92% [22] during follow-up. Finally, the remaining studies [12,13,14,15,17] found no significant differences between interventions [12,14,15] or between subgroups of patients [13,17] based on Kaplan–Meier curves and did not report specific time-based rates, thereby limiting quantitative comparisons between strategies.

### 3.6. ESt Versus Previously Established Interventions

Five studies [12,14,15,20,23] compared the outcomes of ESt with other interventions. Two [14,15] compared ESt with ileocecal resection (ICR) in primary [15] and anastomotic [14] strictures. In both studies, patients undergoing ICR seemed to have greater symptom improvement (90.0% vs. 50.0%, *p* = 0.07 [15] and 83.7% vs. 58.3%, *p* = 0.004 [14]) and a lower need for CD medication escalation (18.8% vs. 23.1%, *p* = 0.74 [15] and 4.1% vs. 17.1%, *p* = 0.005 [14]). However, patients undergoing ESt had less post-procedural complications (6.9% vs. 25.0% per procedure, *p* = 0.05 [15] and 10.2% vs. 31.9% per procedure, *p* = 0.003 [14]) and similar need for subsequent stricture-related surgery (15.4% vs. 18.8%, *p* = 0.79 [15] and 11.3% vs. 10.2% *p* = 0.83 [14]). Likewise, surgery-free survivals (Kaplan–Meier curve: *p* = 0.24 [14], *p* = 0.98 [15]) were comparable between the two procedures (Table A5). In the study assessing anastomotic [14] strictures, propensity score match was performed, resulting in 12 patients in each group. After matching, the rate of symptom improvement was similar between ESt and ICR (60.0% vs. 66.7%, *p* = 0.75) as well as the rate of escalation of medications (8.3% vs. 0.0%, *p* = 0.31). However, emergency department visits (25.0% vs. 33.3% *p* = 0.65), disease-related hospitalizations (33.3% vs. 41.7% *p* = 0.39), rate of subsequent surgery (8.3% vs. 16.7%, *p* = 0.54) and overall morbidity (6.7% vs. 25.0%, *p* = 0.29) were numerically higher in patients receiving ICR.

Three [12,20,25] studies compared the outcomes of ESt with EBD. Ref. [12] reported higher technical (100% vs. 89.5% *p* = 0.25) and clinical (72.7% vs. 45.4, *p* = 0.08) success rates in the ESt group, with similar rates of endoscopic stricture improvement. Most adverse events, including perforation, emergency department visits, hospitalization, escalation of medicine and need for additional endoscopic therapy were numerically lower in the ESt group. However, the bleeding rate was significantly higher with ESt intervention (14.3% vs. 0%, *p* < 0.0001), and there was no statistical difference in surgery-free survival between the interventions (Kaplan–Meier curve, *p* = 0.44), although the need for additional surgery was lower with ESt (9.5% vs. 33.5%, *p* = 0.03). On the other hand, [20] reported ESt was associated with a 54% (adjusted HR 0.46, 95% confidence interval (CI) 0.21–0.99) reduction in the need for repeat endoscopic procedure compared to EBD, a trend towards lower surgery rates than EBD (HR 0.313, 95% CI 0.08–1.18; *p* = 0.08), and a non-significant increase in the rate of significant bleeding was observed with EST (5.9% vs. 1.2%, *p* = 0.18). Notably, the randomized control study [25] found that although technical (88% ESt vs. 88% EBD) and clinical (96% ESt vs. 92% EBD) success were similar, at median 12-month follow-up ESt significantly reduced clinical recurrence (24.5% vs. 54.3%, *p* = 0.003), reintervention (23.5% vs. 52%, *p* = 0.004), emergency visits (17.6% vs. 54%, *p* < 0.001), and hospitalization (15.7% vs. 38%, *p* = 0.01). Surgery was numerically lower (3.9% vs. 16%, *p* = 0.051) as well as the rate of adverse events (13.7% vs. 22%, *p* = 0.31) with ESt. Moreover, Cox regression analysis showed that ES was associated with prolonged time to recurrence (HR 0.35, *p* = 0.004), reintervention (HR 0.36, *p* = 0.006), and emergency department visits (HR 0.28, *p* < 0.001), with a trend towards delayed hospitalization (HR 0.42, *p* = 0.02), but no difference in surgery rates. (Table A6).

Finally, ref. [12] reported outcomes of an additional group of five patients who underwent ESt and EBD at the same session, with 100% technical success, 50% clinical success and 0% adverse events. Similarly, [23] compared the outcomes of ESt with combined therapy (ESt and EBD), reporting fewer adverse events with combined therapy than ESt (10.3% vs. 0%, *p* = 0.87) but greater need for additional endoscopic therapy (38.4% vs. 63.6%, *p* = 0.17) (Table A7).

## 4. Discussion

**Figure 2:**
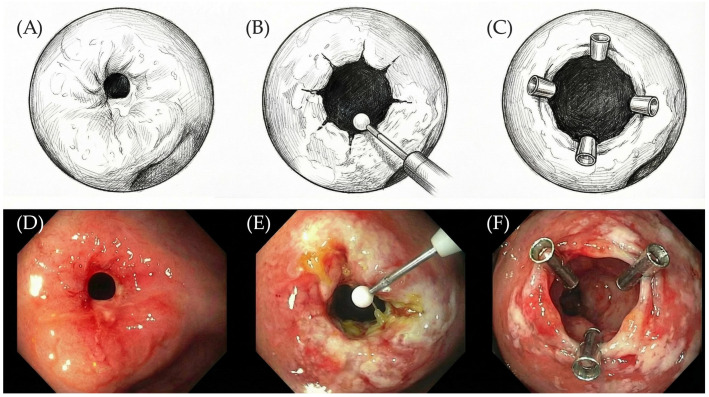
(**A**,**D**) Intestinal stricture. (**B**,**E**) Post-endoscopic stricturotomy. (**C**,**F**) Post-endoscopic strictureplasty. This figure has been created by the authors and is a conceptual illustration of intestinal stricture and its endoscopic treatment. Subfigures (**D**–**F**) are AI-generated representations derived from human images using Nano Banana Pro and have not been previously published.

This systematic review aimed to provide a comprehensive overview of the technical performance and safety of endoscopic stricturotomy and strictureplasty (schematically presented in Figure 2) in the management of CD-associated intestinal strictures.

Optimal management of CD-related strictures relies on appropriate selection of patients and strictures for each therapeutic modality, including surgery, EBD, and ESt/ESTx. Surgery, including segmental resection or Heineke–Mikulicz strictureplasty, remains the standard of care for long (>5 cm), multiple, or complex strictures, particularly in the presence of fistulas, abscesses, or active inflammation, but is associated with recognized risks such as postoperative morbidity, anastomotic recurrence, and, after repeated resections, short bowel syndrome [7,26]. Lan et al. showed that overall morbidity and adverse events rate were higher in patients undergoing ileocecal resection (ICR) than ESt, while the need for subsequent surgery and surgery-free survival were comparable between groups [14,15]. Although symptom improvement was greater and medication escalation rates were lower after ICR, these findings suggest that ESt may delay or potentially obviate the need for surgical resection in a subset of patients with a primary [15] or anastomotic [14] CD-related stricture. On the other hand, EBD is considered a suitable first-line option for patients with clinically significant obstructive symptoms and short (≤5 cm) naïve benign strictures without sharp angulation, deep ulcers, or associated internal fistulas or abscesses [27,28]. The meta-analysis by Bettenworth et al. [6] demonstrated high technical success (94.9%) and symptomatic relief (82.3%), although the risk of major complications (bleeding, perforation or dilation-related surgery) was 5.3%; 38.8% patients required repeat dilation and 27.4% ultimately required surgery during a mean follow-up of 20.5 months. Consistent with these findings, the Global Interventional IBD Group considers EBD safe for both primary and anastomotic strictures but emphasizes that repeated interventions are frequently necessary due to limited long-term clinical success [29].

The high rates of reintervention and progression to surgery following EBD have prompted the development of endoscopic electroincision techniques [28]. ESt and ESTx are emerging as endoscopic options in selected patients with short (<4 cm), primary or secondary straight strictures [9,30,31,32]. These techniques may be preferred over EBD in patients with predominantly fibrotic, tight, or anastomotic strictures [29], particularly when symptoms persist or recur despite repeated dilation, and may represent a logical next step within a step-up endoscopic strategy, bridging conventional dilation and surgery. Indeed, compared to EBD, ESt provides more precise control over incision depth and orientation, making it especially advantageous for benign distal bowel, rectal and anal strictures [29], possibly reducing the risk for perforation [11]. Nevertheless, although its efficacy and safety have been mostly demonstrated for ileocolonic, anorectal or anastomotic strictures, recently Ning et al. [19] have reported that ESt and ESTx are also effective and safe for small bowel strictures. Lan and Shen [12] have shown that ESt is more technically demanding but offers higher technical and clinical efficacy than EBD, lower rates of subsequent surgery, and a lower perforation risk, although the risk of immediate or delayed bleeding is higher. Similarly, Kochar et al. [20] reported fewer repeat endoscopic interventions and possibly fewer surgeries with ESt, with a non-significant increase in clinically relevant bleeding. Additionally, a recent randomized controlled trial with a median follow-up of 12 months [25] showed that, although the technical and clinical success of ESt and EBD were comparable, ESt significantly reduced clinical recurrence, reintervention, emergency department visits and hospitalizations. Moreover, surgery and adverse events rates were numerically lower, and Cox regression analysis further demonstrated that ESt prolonged time to recurrence, reintervention, and emergency visits.

In this review, ESt demonstrated consistently high technical success rates (>88% and often 100%) across studies, whereas clinical success was notably lower, ranging from 50% to 96%, possibly suggesting a gap between technical success and clinical benefit. Even though these endpoints inherently represent distinct aspects of treatment efficacy, they should be interpreted with caution. While technical success is assessed immediately after the endoscopic intervention and is typically defined as successful endoscopic passage through the stricture, clinical success reflects symptomatic improvement and requires sustained maintenance of an adequate luminal diameter. Consequently, factors such as stricture recurrence, persistent inflammation, the severity of fibrosis, and disease progression may contribute to lower clinical success rates despite technically successful procedures. However, identifying which factors are the causes of this gap is challenging, considering the limited number of studies and the available data. Bleeding was the most common adverse event, with rates below 12%, and perforation was rare (mostly <2%). Long-term outcomes varied widely; while some studies reported significant endoscopic reintervention rates (>60%), others reported lower rates (≈18%). Surgical reintervention during follow-up ranged from 0% to 18%. A recent meta-analysis by Jaber et al. [33] analyzed outcomes following ESt in IBD-associated strictures, reporting high technical success (96.4%) and similarly a lower clinical success (62%), with acceptable safety outcomes. These findings are consistent with the overall evidence synthesized in our systematic review. Notably, the present review expands upon previous work by incorporating a broader and more up-to-date body of literature and considering both ESt and ESTx techniques, thereby providing a more comprehensive and contemporary overview of endoscopic electroincision strategies. Nevertheless, long-term outcomes remain insufficiently characterized.

In contrast, ESTx has been evaluated in only three studies, all of which reported high technical (≈92–100%) and clinical (≈71–100%) success rates. Bleeding (≈5–9%) and perforation (0–3%) rates were low and comparable across studies. However, the need for further endoscopic intervention (≈7–57%) and subsequent surgery (≈10–25%) varied widely, highlighting substantial heterogeneity and the limited evidence base. While these preliminary results suggest that ESTx may be a feasible and safe alternative electroincision technique, more robust and long-term data are required to define its comparative effectiveness and durability relative to both ESt and EBD.

Overall, only two studies evaluated clinical recurrence (reoccurrence of initial symptoms), with one reporting no recurrence at 144 ± 105 days and the other reporting 24.5% at one year, and only four studies reported specific time-based probabilities of reintervention-free or surgery-free survival with ESt/ESTx, with reintervention-free rates of approximately 60–64% and surgery-free rates of 75–90% at one year. Greater emphasis on these outcomes is warranted, as symptom recurrence and limited durability are major limitations of EBD, with a cumulative redilatation-free rate of 61.2% and cumulative surgery-free rates of 72.6% at 20.5 months [6]. Whether ESt can meaningfully reduce the need for subsequent endoscopic or surgical interventions therefore remains uncertain. Although Lan [12] found no statistical difference in surgery-free survival between ESt and EBD, despite numerically fewer subsequent surgeries in the ESt group, Kochhar [20] reported a reduction with ESt in the need for repeat endoscopic procedures compared to EBD and a trend towards lower surgery rates. Similarly, Pal [25] demonstrated that ESt significantly reduced clinical recurrence and reintervention. Nevertheless, longer follow-up and comparative studies are required to clarify these potential benefits.

Although current evidence is limited and largely derived from small cohort studies, EST/ESTx are less invasive than surgery, potentially better tolerated and repeatable, and may delay or reduce the need for surgical intervention without precluding subsequent surgery. Nevertheless, patients undergoing Est/ESTx should be monitored closely, and a backup strategy, such as endoscopic clipping or surgical intervention, should be readily available whenever needed [11,19]. Lastly, whereas most studies have reviewed Est/ESTx and EBD separately, some have explored their combined use [23,34]. A recent randomized controlled trial [34] explored the benefit of combining ESt with multiple EBD sessions. In this study, patients who underwent more intensive endoscopic interventions experienced greater symptomatic improvement compared with less intensive strategies, suggesting that multimodal endoscopic approaches may warrant further investigation.

This review is limited by the small number of studies and CD patients subjected to ESt/ESTx, heterogeneity of patient’s underlying disease conditions and previous treatments (EBD or surgery), short follow-up periods, heterogeneity in outcome measurements, and incomplete reporting (with some studies not reporting clinical success and some adverse events), particularly in abstract-only publications, possibly introducing publication bias. Abstract-only publications provide limited methodological detail and less comprehensive reporting, potentially contributing to heterogeneity in pooled analyses. Nevertheless, exploratory subgroup analyses—limited by the small number of studies—demonstrated broadly consistent effectiveness and safety outcomes regardless of study format or endoscopic technique. Also, the exclusion of case series with fewer than 10 patients may have resulted in the omission of early safety data and introduced potential bias. Although one study used a multicentre design, most studies were conducted in tertiary referral centres, potentially introducing referral and selection bias, and several studies originate from a limited number of specialized centres with expertise in advanced endoscopic management of CD-related strictures, potentially contributing to a centre effect. Additionally, in most studies, the procedure was consistently performed by a single endoscopist. While this may ensure technical consistency, it raises concerns regarding the reproducibility of the technique and the generalizability of the results. These factors highlight the need for future multicentre prospective studies involving a broader range of institutions to better validate the reproducibility and generalizability of these techniques. All studies relied on medical records to assess intervention and outcomes, resulting in a relatively low risk of misclassification bias. Nevertheless, it is important to notice that the decision to treat the patient with ESt was at the discretion of treating endoscopists and usually based on clinical factors such as short length (<5 cm) and absence of severe inflammation, possibly introducing indication/selection bias. Therefore, the comparative outcomes between ESt, EBD, and surgical interventions should be interpreted with caution, since most available studies are non-randomized and subject to confounding by indication. Accordingly, these comparative results should be considered hypothesis-generating rather than definitive evidence of superiority or equivalence. Moreover, a significant proportion of patients were submitted to EBD before or simultaneously with ESt, and several studies combined data from ESt and ESTx without differentiating between the two techniques, despite potential differences in efficacy and safety. Additionally, the definition of EStx was not always clearly specified. This represents a major limitation, as pooling data from studies with mixed or poorly defined interventions may introduce substantial heterogeneity and make outcomes attributed to EStx difficult to interpret, potentially conflating them with those of ESt alone. Finally, while technical success is relatively simple to assess, clinical improvement is inherently subjective. As acknowledged by some studies [16,20], relying on patient’s symptoms alone to assess the success of ESt also lends subjectivity, as sometimes symptoms do not correlate with stricture severity [35]. Hence, defining clinical success primarily based on symptom improvement is a limitation. However, this reflects the outcome definitions reported in the included studies and the inconsistent use of more objective measures, such as endoscopic reassessment or imaging, highlighting the need for future studies to adopt more standardized and objective endpoints to better assess the true clinical impact of these techniques.

Despite these limitations, this review provides an important contribution to a rapidly evolving field. By incorporating the most recent evidence available up to 2025, including the only randomized controlled trial published to date, it offers an updated synthesis of the safety and efficacy of ESt/ESTx in CD-associated strictures. Overall, ESt and ESTx appear particularly beneficial for short, straight and accessible fibrotic strictures, particularly if anastomotic or refractory to EBD. These techniques represent an evolution toward a form of endoluminal surgery, allowing targeted treatment of fibrostenotic disease while preserving bowel length. However, their use requires advanced endoscopic expertise and should be confined to experienced centres. These findings support the expanding role of therapeutic endoscopy in IBD, reflecting a paradigm shift from purely diagnostic procedures toward advanced endoluminal interventions that may bridge the gap between medical therapy and surgery.

Standardized definitions of technical and clinical success, along with prospective, multicentre studies with larger patient populations and longer follow-up, are needed to refine patient selection, better define safety profiles, and establish the precise role of ESt and ESTx within the treatment algorithm for CD-related strictures. Notably, one ongoing randomized controlled trial, DESTRESS, is assessing surgery and endoscopic-free survival, technical and clinical efficacy, and safety over a 1-year follow-up of EBD versus ESt in patients with CD-associated strictures (NCT05009212).

## Figures and Tables

**Table 2:** Description of the endoscopic procedures.

Study ID	ESt	ESTx
Lan, 2017 USA [11]	Material: regular needle knife or needle knife with isolated ceramic tip in the setting of electroincision or electrocauterization at the discretion of the endoscopist. Technique: NR All procedures were performed by the same endoscopist (B.S.).	-
Lan, 2018 USA [12]	Material: triple- lumen needle knife (Boston Scientific, Marlborough, MA, USA) or a single-use electrosurgical IT knife2 (Olympus Medical Systems, Tokyo, Japan) under the setting of ERCP Endocut on ERBE (USA Incorporated Surgical Systems, Marietta, GA, USA). Technique: strictures were incised in a circumferential or radial fashion until an adequate passage of the scope. All procedures were performed by the same endoscopist (B.S.).	Technique: endoclips were deployed after radial cut to keep treated stricture open and were also used in controlling bleeding for both radial and circumferential cuts.
Lan, 2019 USA [13]	NR	NR
Lan, Stocchi, 2019 USA, China [14]	Material: triple-lumen needle knife (Boston Scientific, Marlborough, MA, USA) or a single-use electrosurgical IT knife 2 (Olympus Medical Systems, Tokyo, Japan) under the setting of ERCP Endocut (Erbe USA Incorporated Surgical Systems, Marietta, GA, USA). Technique: strictures were incised in a circumferential or radial manner until adequate passage of the endoscope was achieved. All procedures were performed by the same endoscopist (B.S.).	-
Lan, 2020 USA [15]	Material: Boston Scientific triple-lumen needle knife (Boston Scientific, Marlborough, MA, USA) or Olympus single-use electrosurgical IT knife 2 (Olympus Medical Systems, Tokyo, Japan) under the setting of ERCP Endocut (ERBE USA Incorporated Surgical Systems, Marietta, GA, USA). Technique: strictures were incised in a circumferential or radial manner until the adequate passage of the scope was achieved. All procedures were performed by the same endoscopist (B.S.).	-
Mohy-Ud-Din, 2020 USA [16]	Material: endoscopic Nano knife (Olympus Medical Systems, Tokyo, Japan) with the current setting Endocut-I (ERBE USA, Marietta, GA, USA). Technique: strictures were cut in either radial, horizontal, semicircumferential, or circumferential fashion. All procedures were performed by one endoscopist (G.K.).	Material: Through-the-scope (TTS) clips. Technique: TTS clips deployed at the site of incision in circumferential fashion to prevent the incised edges from coalescing back together, preventing restricture formation, and also helping prevent any delayed bleeding.
Zhang, 2020 USA [17]	Material: Boston Scientific triple-lumen needle knife (Boston Scientific, Marlborough, MA, USA) or Olympus single-use electrosurgical IT knife 2 (Olympus Medical Systems, Tokyo, Japan) under the setting of ERCP Endocut (ERBE USA Incorporated Surgical Systems, Marietta, GA, USA). Technique: strictures were incised in a circumferential or radial manner until adequate passage of the scope was achieved. All procedures were performed by one endoscopist (B.S.).	-
Lukas, 2022 Czech Republic [18]	NR	NR
Ning, 2023 China [19]	Material: double-balloon enteroscope (EN-450P5 or EN-580T; Fujifilm, Tokyo, Japan) or a single-balloon enteroscope (SIF-Q260; Olympus Medical Systems, Tokyo, Japan); hook knife (KD-620UR, Olympus Medical Systems, Tokyo, Japan) or IT knife nano (KD-612 U, Olympus Medical Systems, Tokyo, Japan) with an Erbe machine (Erbe Elektromedizin GmbH, Tuebingen, Germany) using the ESD endoCUT Q setting. Technique: strictures were incised in a radial or circumferential fashion until the scope passed. The procedures were performed by one of the three endoscopists (Q.G., S.N., and Y.Z.).	Material: endoclips, argon plasma coagulation (Erbe Elektromedizin GmbH, Tuebingen, Germany), or high-frequency hemostatic forceps (FD-411UR; Olympus Medical Systems, Tokyo, Japan) were used at the discretion of the endoscopist for hemostasis.
Kochhar, 2023, USA [20]	Material: NR. Technique: NR All procedures were performed by one endoscopist.	-
Khan, 2024 USA [21]	NR	NR
Herman, 2024 USA [22]	Material: upper endoscope (GIF series, Olympus, Tokyo); soft-tip guidewire was used in selected patients with pinhole strictures or adjacent fistulae; IT, IT2 (Olympus, Tokyo), NK (Boston Scientific, 300 Boston Scientific Way Marlborough, MA, USA) and Erbe VIO 300Delectrosurgical generator (ERBE USA, Marietta, GA, USA) with a setting of Endocut mode were used to perform electroincision and/or electrocautery treatment. Technique: NR All procedures were performed by a single endoscopist (B.S.).	-
Chaudhary, 2024, USA [23]	NR	NR
Cui, 2025 China [24]	Material: single-balloon enteroscope (SIF-Q260, Olympus) and IT knife (KD-611 L; Olympus) Technique: radial incisions were made at the stricture site from shallow to deep under direct visualization. During the procedure, the following precautions were taken: avoided overinflation, controlled the depth of the incision, preserved as much of the muscle layer as possible, and examined the incision site for postoperative bleeding and perforation. All procedures were performed by a single endoscopist (senior chief physician).	-
Pal, 2026, India [25]	NR	NR

ESt, stricturotomy; ESTx, strictureplasty; -, not performed; NR, not reported.

**Table 3:** Technical and clinical outcomes.

Study ID	Technical Success	Clinical Success	Endoscopic Stricture Improvement	Technical Success Definition	Clinical Success Definition
Lan, 2017, USA [11]	85/85 (100%) patients	29/53 (54.7%) patients	NR	Passage of the gastroscope or pediatric colonoscope without resistance.	Symptomatic improvement.
Lan, 2018, USA * [12]	21/21 (100%) patients	8/11 (72.7%) patients	8/17 (47.1%) patients	Passage of endoscope without resistance.	Symptomatic improvement.
Lan, 2019, USA [13]	Primary stricture: 45 (95.7%) Anastomotic strictures: 36 (97.3%)	Primary stricture: 15 (46.9%) Anastomotic strictures: 11 (50.0%)	Primary stricture: 15 (35.7%) Anastomotic strictures: 6 (22.2%)	NR	Symptomatic improvement.
Lan, Stocchi, 2019, USA, China [14]	34/35 (97.1%) patients	14/24 (58.3%) patients	NR	Passage of endoscope without resistance.	Symptomatic improvement.
Lan, 2020, USA [15]	13/13 (100%) patients	3/6 (50.0%) patients	6/13 (46.2%) patients	Passage of endoscope without resistance.	Symptomatic improvement.
Mohy-Ud-Din, 2020, USA * [16]	11/12 (92%) procedures	10/11 (91%) patients	NR	Traversability of the scope without resistance.	Symptom improvement.
Zhang, 2020, USA [17]	49/49 (100%) patients	23/34 (67.6%) patients	10/49 (20.4%) patients	Passage of the pediatric colonoscope without resistance.	Symptomatic improvement.
Lukas, 2022, Czech Republic [18]	83 (90.2%) procedures	NR	NR	Ability to pass the scope through the stricture following the procedure.	NR
Ning, 2023, China * [19]	26/28 (92.9%) patients 56/58 (96%) procedures	Short-term: 20/28 (71.4%) patients; long-term: (74.8%)	NR	Ability to pass the scope beyond the stricture after the procedure.	Short-term: the improvement of symptoms at week 8. Long-term: surgery-free rate at 1 year of follow-up.
Kochhar, 2023, USA [20]	NR	NR	NR	NR	NR
Khan, 2024, USA [21]	NR	NR	NR	NR	NR
Herman, 2024, USA [22]	24/24 (100%) patients	NR	NR	Ability to traverse the strictured site with the endoscope.	NR
Chaudhary, 2024, USA [23]	NR	NR	NR	NR	NR
Cui, 2025, China [24]	11/11 (100%) patients	NR	NR	Successful incision of the stricture lesion, allowing the enteroscope to pass smoothly through the stricture.	NR
Pal, 2026, India [25]	45/51 (88%) patients	49/51 (96%) patients	NR	Ability to pass pediatric colonoscope beyond stricture.	Reduction in CD obstruction Score (CDOS) **≥** 1-point drop.

NR, not reported. * The intervention was ESt and ESTx.

**Table 4:** Adverse events reported in the different studies.

Study ID	Adverse Events	Bleeding	Perforation	Need for Additional Endoscopic Procedures **	Subsequent Surgery	Stricture-Related ER Visit	Stricture-Related Hospitalization	Escalation of Medication
Lan, 2017, USA [11]	10/272 (3.7%) procedures	9/85 (10.6%) patients	1/85 (1.2%) patients	77/127 (60.6%) strictures: 57/127 (44.9%) ESt 14/127 (11.0%) combined EBD + ESt 29/127 (22.8%) EBD	13/85 (15.3%) patients	11/85 (12.9%) patients	18/85 (21.2%) patients	NR
Lan, 2018, USA * [12]	NR	3/21 (14.3%) patients; 4/45 (8.8%) procedures	0/21 (0%) patients; 0/45 (0%) procedures	12 (57.1%) patients: 6 ESt, 4 EBD and 2 combined EBD + ESt	2/21 (9.5%) patients	2/21 (9.5%) patients	1/21 (4.8%) patients	3/21 (14.3%) patients
Lan, 2019, USA [13]	Primary strictures: 5 (5.4%); secondary (anastomotic) strictures: 3 (4.2%)	NR	NR	NR	Primary strictures: 7 (14.9%); secondary strictures: 8 (21.6%)	NR	NR	NR
Lan, Stocchi, 2019, USA, China [14]	5/49 (10.2%) procedures	4/49 (8.2%) procedures	1/49 (2%) procedures	NR	4 (11.3%) patients	Disease-related: 8/35 (22.9%) patients	Disease-related: 8/35 (22.9%) patients	6/35 (17.1%) patients
Lan, 2020, USA [15]	2/29 (6.9%) procedures	NR	2/13 *** (15.4%) patients	NR	2/13 (15.4%) patients	0/13 (0%) patients	5/13 (38.5%) patients	3/13 (23.1%) patients
Mohy-Ud-Din, 2020, USA * [16]	NR	1/11 (9%) patients	0/11 (0%) patients	4/12 (33%) strictures EBD right after Est (same session); 1/12 (8%) strictures ESt in different sessions	NR	NR	1/11 (9%) patients	NR
Zhang, 2020, USA [17]	NR	5/106 (4.7%) procedures	0/49 (0%) patients	NR	6/49 (12.2%) patients	Disease-related: 7/49 (14.3%) patients	Disease-related: 10/49 (20.4%) patients	NR
Lukas, 2022, Czech Republic [18]	4/67 (6%) patients	4/67 (6%) patients	NR	NR	NR	NR	NR	NR
Ning, 2023, China * [19]	5/58 (8.6%) procedures	3/58 (5.2%) procedures	2/58 (3.4%) procedures	2/28 (7.1%) patients	7/28 (25%) patients	NR	NR	NR
Kochhar, 2023, USA [20]	NR	4/68 (5.9%) patients	0/0 (0%) patients	NR	NR	NR	NR	NR
Khan, 2024, USA [21]	0/48 (0%) patients	NR	NR	24/48 (50.0%) patients	0/48 (0%) patients	NR	NR	NR
Herman, 2024, USA [22]	0/24 (0%) patients	0/24 (0%) patients	0/24 (0%) patients	16/24 (66.6%) ESt; 1/24 (4.2%) ESt and surgical reintervention	2/24 (8.3%) patients	NR	NR	NR
Chaudhary, 2024, USA [23]	4/39 (10.3%) patients	3/39 (7.7%) patients	NR	15/39 (38.4%) patients	1/39 (2.5%) patients	NR	NR	NR
Cui, 2025, China [24]	0/11 (0%) patients	0/11 (0%) patients	0/11 (0%) patients	2/11 (18.2%) patients	2/11 (18.2%) patients	NR	NR	NR
Pal, 2026, India [25]	7/51 (13.7%) patients	6/51 (11.8%) patients	1 (2.0%) patients	12/51 (23.5%) patients	2/51 (3.9%) patients	9/51 (17.6%) patients	8/51 (15.7%) patients	NR

* The intervention was ESt and ESTx. ** The need to repeat endoscopic treatment during the follow-up period after initial ESt, with additional ESt, EBD, or both. ESt, stricturotomy; EBD, endoscopic balloon dilation. NR, not reported. *** 1 of these 2 patients was suspected, not confirmed.

## Data Availability

No new data were created or analyzed in this study. Figure 2 is a conceptual illustration of intestinal stricture and its endoscopic treatment, was partially generated by the authors using artificial intelligence tools, and has not been previously published.

## Abbreviations

The following abbreviations are used in this manuscript:

CD	Crohn’s disease
IBD	Inflammatory bowel disease
ESt	Endoscopic stricturotomy
ESTx	Endoscopic stricturoplasty
EBD	Endoscopic balloon dilation
ICR	Ileocecal resection
AS	Anastomotic strictures
NAS	Non-anastomotic strictures

## Appendix A

### Appendix A.2

**Table A1:** Search terms used for each database.

PubMed	(“endoscopic stricturotomy” OR “endoscopic strictureplasty” OR “endoscopic stricturoplasty” OR “endoscopic strictur*” OR “endoscopic electroincision” OR “needle knife” OR “endoscopic incision” OR “ESt” OR “ESTx”) AND (“IBD” OR “inflammatory bowel diseas*” OR “Crohn*” OR “ulcerative colitis”)
Web of Science	
Scopus	

### Appendix A.3

**Table A2:** Characteristics of the patients included in the included studies.

Study ID	Patients		Preprocedure Treatments									
	N.	Disease Phenotype and Location	Pharmacotherapy								Endoscopic	Surgical
			None	5-ASA	Steroids	Anti-TNF	Other Biologics	Immunomodulators	ATB	Others	EBD	
Lan, 2017, USA [11]	n = 85 (85 with IBD, 35 with CD)	L1: 32 (37.6%) L2: 3 (3.5%)				Anti-TNF or ant integrin biologics: 23 (27.1%)					69 (54.3%) of strictures (n= 127)	77/85 (90.6%)
Lan, 2018, USA [12]	n = 185 (21 in the ESt arm: 21 with IBD, 21 with CD)	NR				9 (42.9%)					12/21 (57.1%)	
Lan, 2019, USA [13]	NR	NR										
Lan, Stocchi, 2019, USA, China [14]	n =182 (35 in the ESt arm: 35 with IBD, 35 with CD)	Perianal disease: 8 patients (22.9%)		12 (34.3%)	12 (34.3%)	18 (51.4%)		13 (37.1%)	1 (2.9%)		8/35 (22.9%)	1.0 (range: 1.0–2.0)
Lan, 2020, USA [15]	n = 45 (13 in the ESt arm: 13 with IBD, 13 with CD)	L1 or L3 B2: 13 (100%) Perianal disease: 4 (30.8%)		6 (46.2%)	4 (30.8%)	5 (38.5%)		5 (38.5%)				
Mohy-Ud-Din, 2020, USA [16]	n = 11 (11 with IBD, 7 with CD)	L2: 2 L3: 5	3 (27%)			Adalimumab: 2 (18%) IFX: 1 (9%)	Vedolizumab: 4 (36%) Ustekinumab: 1 (9%)				2 (17%) of strictures (n = 12)	10/11 (91%)
Zhang, 2020, USA [17]	n = 64 (49 with IBD, 25 with CD)	NR		8 (16.3%)	9 (18.4%)	13 (26.5%)		15 (30.6%)	13 (26.5%)	NSAIDS:1 (2.0%)	25/49 (51.0%)	
Lukas, 2022, Czech Republic [18]	n = 67 (67 with IBD, 60 with CD)	NR									53.3% of the procedures (ESt: 27.2% of the procedures)	
Ning, 2023, China [19]	n = 28 (28 with IBD, 28 with CD)	L1: 24 (85.7%) L3: 4 (14.3%) B2: 25 (89.3%) B3: 3 (10.7%) Perianal: 4 (14.3%)		1 (3.6%)	1 (3.6%)	IFX: 1 (3.6%)				IFX + AZA/MTX: 3 (10.7%) MTX: 1 (3.6%)		1/28 (3.6%)
Kochhar, 2023, USA [20]	n = 149 (68 in the ESt arm: 68 with IBD, 68 with CD)	NR			7 (10%)	29/52 (56%)	Vedolizumab: 10/52 (19%) Ustekinumab: 13/52 (25%)					43/68 (63%)
Khan, 2024, USA [21]	n = 48 (48 with IBD, 48 with CD)	NR			NAS patients: 4 (22.2%) AS patients: 7 (23.3)							
Herman, 2024, USA [22]	n = 24 (24 with IBD, 18 with CD)	NR			Budesonide: 2 (8%) Prednisone: 2 (8%)	Adalimumab: 2 (8%) IFX: 4 (16%)	Ustekinumab: 7 (29%) Vedolizumab: 3 (12.5%)			AZA: 3 (12.5%) MTX:1 (4%)	3/24 (12.5%)	
Chaudhary, 2024, USA [23]	n = 50 (39 in the ESt arm: 39 with IBD, NR with CD)	NR	9 (23%)	Balsalazide: 1 (2.5%) Mesalamine: 3 (7.6%)	Budesonide: 2 (5.1%) Prednisone: 3 (7.6%)	Adalimumab: 7 (17.9%) IFX: 4 (10.2%)	Ustekinumab: 4 (10.2%) Vedolizumab: 3 (7.6%) Risankizumab: 5 (12.8%) Romosozumab: 1 (2.5%)	Upadacitinib: 2 (5.1%)		AZA: 4 (10%)		
Cui, 2025, China [24]	n = 11 (11 with IBD, 11 with CD)	NR										3/11 (27.3%)
Pal, 2026, India [25]	n = 151 (51 in the ESt arm: 51 with IBD, 51 with CD)	L1: 7 (13.7%) L2: 18 (35.3%) L3: 24 (47.1%) L4: 2 (3.9%) B2: 45 (88.2%) B2+B3: 6 (11.8%) Perianal: 5 (9.8%)		3 (5.9%)	4 (7.8%)	Adalimumab: 9 (17.6%) IFX: 6 (11.8%)	Ustekinumab: 5 (9.8%)			AZA: 41 (80.4%) MTX: 2 (3.9%)	4 (7.8%)	

5-ASA, aminosalicylic acid; AS, anastomotic; AZA, 6-mercaptopurine/azathioprine; IFX, infliximab; anti-TNF, anti-tumour necrosis factor; MTX, methotrexate; NAS, non-anastomotic; NSAIDS, non-steroidal anti-inflammatory drug; NR, not reported.

### Appendix A.4

**Table A3:** CASP (Critical Appraisal Skills Programme) checklist summarizing the appraisal of methodological quality across the included studies. Each item reflects key domains such as study validity, results, and applicability. Six of the included studies (*) were available only in abstract form.

	CASP Checklist			Ning, 2023 [19]	Lan, 2020 [15]	Lukas, 2022 [18] *	Zhang, 2019 [17]	Lan, 2019 [13] *	Mohy-Ud-Din, 2020 [16]	Kochhar, 2023 [20] *	Lan, 2018 [12]	Lan, Stocchi, 2019 [14]	Lan, 2017 [11]	Herman, 2024 [22]	Khan, 2024 [21] *	Chaudhary, 2024 [23] *	Cui, 2025 [24]	Pal, 2026 [25] *
Section A: Are the results valid?		1. Did the study address a clearly focused issue?		●	●	●	●	●	●	●	●	●	●	●	●	●	●	●
		2. Was the cohort recruited in an acceptable way?		●	●	●	●	●	●	●	●	●	●	●	●	●	●	●
		3. Was the exposure accurately measured to minimize bias?		●	●	●	●	●	●	●	●	●	●	●	●	●	●	●
		4. Was the outcome accurately measured to minimize bias?		●	●	●	●	●	●	●	●	●	●	●	●	●	●	●
		5. Confounding factors:	(a) Have the authors identified all important confounding factors?	●	●	●	●	●	●	●	●	●	●	●	●	●	●	●
			(b) Have they taken account of the confounding factors in the design/analysis?	●	●	●	●	●	●	●	●	●	●	●	●	●	●	●
		6. Follow up:	(a) Was the follow up of subjects complete enough?	●	●	●	●	●	●	●	●	●	●	●	●	●	●	●
			(b) Was the follow up of subjects long enough?	●	●	●	●	●	●	●	●	●	●	●	●	●	●	●
Section B: What are the results?		7. What are the results of this study?		●	●	●	●	●	●	●	●	●	●	●	●	●	●	●
		8. How precise are the results?		●	●	●	●	●	●	●	●	●	●	●	●	●	●	●
		9. Do you believe the results?		●	●	●	●	●	●	●	●	●	●	●	●	●	●	●
Section C: Will the results help locally?		10.Can the results be applied to the local population?		●	●	●	●	●	●	●	●	●	●	●	●	●	●	●
		11.Do the results of this study fit with other available evidence?		●	●	●	●	●	●	●	●	●	●	●	●	●	●	●
		12.What are the implications of this study for practice?		●	●	●	●	●	●	●	●	●	●	●	●	●	●	●

Yes (●), No (●), Can’t tell (●) | * Study only published in the abstract form.

### Appendix A.5

**Table A4:** Random-effects meta-analysis of proportions for each outcome—pooled estimates with 95% confidence intervals (CIs) are shown. Subgroup analyses were performed according to study type (abstract vs full-text) and endoscopic technique (ESt, ESt + ESTx, ESt + EBD); *p*-values represent tests for subgroup differences.

Outcome	Analysis	Subgroup	Subgroup Category	Studies	Pooled Proportion (95% CI)	Heterogeneity (I^2^)	Heterogeneity (τ^2^)	Subgroup Differences (*p*-Value)
Technical success	Overall	-	-	12	0.93 (0.89 to 0.96)	13.5%	0.08	-
	Subgroup	Study type	Full text	9	0.95 (0.90 to 0.97)	0.0%	0.00	0.21
			Abstract	3	0.91 (0.71 to 0.98)	38.6%	0.08	
		Technique	ESt	8	0.94 (0.87 to 0.97)	38.7%	0.25	0.80
			ESt + ESTx	3	0.92 (0.70 to 0.98)	0.0%	0.00	
			ESt + EBD	1	0.96 (0.58 to 0.99)	-	-	
Clinical success	Overall	-		9	0.68 (0.51 to 0.81)	68.6%	0.52	-
	Subgroup	Study type	Full text	7	0.63 (0.53 to 0.72)	4.5%	0.01	0.57
			Abstract	2	0.80 (0 to 1)	94.7%	4.43	
		Technique	ESt	6	0.65 (0.38 to 0.85)	75.7%	0.80	0.42
			ESt + ESTx	3	0.736 (0.48 to 0.89)	0.0%	0.00	
Endoscopic stricture improvement	Overall	-		4	0.33 (0.17 to 0.55)	49.1%	0.15	-
	Subgroup	Study type	Full text	3	0.36 (0.09 to 0.77)	65.8%	0.35	0.65
			Abstract	1	0.31 (0.21 to 0.42)	-	-	
		Technique	ESt + ESTx	1	0.47 (0.26 to 0.69)	-	-	0.18
			ESt	3	0.30 (0.11 to 0.60)	44.4%	0.09	
Need for subsequent surgery	Overall	-		12	0.15 (0.11 to 0.20)	15.0%	0.00	-
	Subgroup	Study type	Full text	8	0.16 (0.12 to 0.20)	0.0%	0.00	0.17
			Abstract	4	0.08 (0.01 to 0.35)	65.2%	0.78	
		Technique	ESt	8	0.14 (0.11 to 0.19)	0.0%	0.00	0.63
			ESt + ESTx	2	0.20 (0.01 to 0.99)	37.0%	0.19	
			ESt + EBD	2	0.06 (0.00 to 1.00)	75.9%	3.98	
Adverse events	Overall	-		15	0.09 (0.07 to 0.12)	0.0%	0.01	-
	Subgroup	Study type	Full text	9	0.11 (0.09 to 0.14)	0.0%	0.00	0.17
			Abstract	6	0.08 (0.04 to 0.14)	35.6%	0.10	
		Technique	ESt	10	0.09 (0.07 to 0.12)	0.0%	0.03	0.06
			ESt + ESTx	3	0.12 (0.05 to 0.23)	0.0%	0.00	
			ESt + EBD	2	0.02 (0.00 to 0.99)	0.0%	0.00	

Abbreviations: CI, confidence interval; ESt, endoscopic stricturotomy; ESTx, extended stricturotomy; EBD, endoscopic balloon dilation.

### Appendix A.6

**Table A5:** Comparison of endoscopic stricturotomy (ESt) with ileocecal resection (ICR).

ESt vs. ICR							
		Lan, Stocchi, 2019 [14]			Lan, 2020 [15]		
		ESt	ICR	*p*-value	ESt	ICR	*p*-value
	Follow-up	0.8 year (IQR 0.2–1.7)	2.2 year (IQR 1.2–4.4)	*p* < 0.001	1.8 year (IQR 1.1–2.4)	1.5 year (IQR 0.8–4.1)	*p* = 0.84
Patient’s characteristics	N	35/182	147/182		13/45	32/45	
	CD	35/35	147/147		13/13	32/32	
	Symptomatic	30/35 (85.7%)	147 (100%)	*p* <0.0001	9/11 (81.8%)	30/30 (100%)	*p* = 0.91
	Previous treatment	8/35 (22.9%) EBD	46/147 (31.3%) EBD	*p* = 0.33	NR	NR	
Stricture’s characteristics	Type	Anastomotic	Anastomotic		Primary	Primary	
	Length	2.0cm (IQR 1.5–2.5)	7.0cm (IQR 4.0–10.0)	*p* <0.001	2.4 ± 0.9 cm (SD)	3.0 ± 1.1 cm (SD)	*p* = 0.17
	Non-traversable	21/31 (67.7%)	49/92 (53.3%)	*p* = 0.36	13/13 (100%)	11/15 (73.3%)	*p* = 0.26
	Location	Ileocolonic	Ileocolonic		Distal ileum	Distal ileum	
Efficacy outcomes	Technical success	34/35 (97.1%)	NR		13/13 (100%)	32/32 (100%)	NR
	Clinical success	14/24 (58.3%) patients	123/147 (83.7%)	*p* = 0.004	3/6 (50.0%) patients	27/30 (90.0%) patients	*p* = 0.07
	Endoscopic stricture improvement	NR	NR		6/13 (46.2%)	32/32 (100%)	*p* <0.001
Adverse events	Adverse Events	5/49 (10.2%) procedures	47/147 (31.9%)	*p* = 0.003	2/29 (6.9%) procedures	8/32 (25.0%) patients	*p* = 0.05
	Bleeding	4/49 (8.2%) procedures	NA		NR	NR	
	Perforation	1/49 (2%) procedures	NA		2/13 (15.4%) patients	NR	
	Additional Est/EBD	NR	NR		NR	NR	
	Additional surgery	4/35 (11.3%)	15/147 (10.2%)	*p* = 0.83	2/13 (15.4%)	6/32 (18.8%)	*p* = 0.79
	Stricture-related ER visit	Disease-related: 8/35 (22.9%)	Disease-related: 46/147(31.3%)	*p* = 0.10	0/13 (0%)	3/32 (9.4%)	*p* = 0.25
	Stricture-related ER hospitalization	Disease-related: 8/35 (22.9%)	Disease-related: 70/147 (47.6%)	*p* = 0.008	5/13 (38.5%)	5/32 (15.6%)	*p* = 0.09
	Escalation of medication	6/35 (17.1%)	6/147 (4.1%)	*p* = 0.005	3/13 (23.1%)	6/32 (18.8%)	*p* = 0.74

### Appendix A.7

**Table A6:** Comparison of endoscopic stricturotomy (ESt) with endoscopic ballon dilations (EBD).

ESt vs. EBD										
		Lan, 2018 [12]			Kochar, 2023 [20]			Pal, 2026 [25]		
		ESt	EBD	*p*-Value	ESt	EBD	*p*-Value	ESt	EBD	*p*-Value
	Follow-up	0.8 year (IQR 0.1–1.6)	4.0 year (IQR 0.8–6.9)	*p* < 0.0001	1.1 year (mean)	1.1 year (mean)		1 year (IQR 0.25–3)	1 year (IQR 0.25–3)	
Patient’s characteristics	N	21/185	164/185		68/149	81/149		51/101	50/101	
	CD	21/21	164/164		68/68	81/81		51/51	50/50	
	Symptomatic	15/21 (71.4%)	135/164 (82.3%)	*p* = 0.23	NR	NR		51/51	50/50	
	Previous treatment	12/21 (57.1%) EBD	NR		43/68 (63%) surgery	52/81 (65%) surgery	*p* = 0.86	4/51 (7.8%) endotherapy	4/51 (8%) endotherapy	*p* = 0.73
Stricture’s characteristics	Type	Anastomotic	Anastomotic		Primary: 44/68 (64.7%) Anastomotic: 24/68 (35.3%)	Primary: 48/81 (59%) Anastomotic: 33/81 (41%)	*p* = 0.61	Primary: 43 (84.3%) Anastomotic: 8 (15.7%)	Primary: 44 (88%) Anastomotic: 6 (12%)	*p* = 0.8
	Length	1.5 cm (IQR 1.0–2.4)	2.0 cm (IQR 1.0–3.0)	*p* = 0.13	NR	NR		1.5 cm (IQR 0.5-3.0)	1.5 cm (IQR 1.0-2.8)	*p* = 0.21
	Non-traversable	12/21 (57.1%)	85 (52.1%)	*p* = 0.36	NR	NR		51 (100%)	51 (100%)	
	Location	Ileocolonic: 18 (85.7%) Ileorectal: 2 (9.5%) Colocolonic: 1 (4.8%)	Ileocolonic: 161 (98.2%) Ileorectal: 2 (1.2%) Colocolonic: 1 (0.6%)	*p* = 0.02	Small bowel: 29/68 (42.6%) Colonic: 39/68 (57.4%)	Small bowel: 44/81 (54.3%) Colonic: 37/81 (45.7%)	*p* = 0.19	Upper GI: 2 (3.9%) Ileal: 10 (19.6%) Ileocecal: 13 (25.5%) Ileal + ileocecal: 3 (5.9%) Colonic: 14 (27.5%) Anorectal: 9 (17.6%)	Upper GI: 3 (6%) Ileal: 17 (34%) Ileocecal: 8 (16%) Ileal + ileocecal: 1 (2%) Colonic: 9 (18%) Anorectal: 11 (22%) Colonic + anorectal: 1 (2%)	*p* = 0.43
Efficacy outcomes	Technical success	21/21 (100%)	147/164 (89.5%)	*p* = 0.25	NR	NR		45/51 (88%)	44/50 (88%)	
	Clinical success	8/11 (72.7%) patients	59/103 (45.4%) patients	*p* = 0.08	NR	NR		49/51 (96%)	46/50 (92%)	
	Endoscopic stricture improvement	8/17 (47.1%) patients	57/163 (35.0%) patients	*p* = 0.32	NR	NR		NR	NR	
Adverse events	Adverse Events	NR	NR		NR	NR		7/51 (13.7%)	11/50 (22%)	*p* = 0.31
	Bleeding	3/21 (14.3%) 4/45 (8.8%) procedures	0/164 (0%) 0/478 (0%) procedures	*p* <0.0001	4/68 (5.9%) patients	1/81 (1.2%)	*p* = 0.18	6/51 (11.8%)	4/51 (8%)	
	Perforation	0/21 (0%)	4/164 (2.4%) 5/478 (1.1%) procedures	*p* = 1.0	0/0 (0%)	0/0 (0%)		1 (2%)	1 (2%)	
	Additional Est/EBD	12/21 (57.1%)	98/164 (59.8%) EBD	*p* = 0.85	NR	NR		12/51 (23.5%)	26/50 (52%)	*p* = 0.004
	Additional Surgery	2/21 (9.5%)	55/164 (33.5%)	*p* = 0.03	NR	NR		2/51 (3.9%)	8/50 (16%)	*p* = 0.051
	Stricture-related ER visit	2/21 (9.5%)	34/164 (20.7%)	*p* = 0.33	NR	NR		9/51 (17.6%)	27/50 (54%)	*p* < 0.001
	Stricture-related ER hospitalization	1/21 (4.8%)	33/164 (20.1%)	*p* = 0.16	NR	NR		8/51 (15.7%)	19/50 (38%)	*p* = 0.01
	Escalation of medication	3/21 (14.3%)	53/164 (32.3%)	*p* = 0.09	NR	NR		NR	NR	

### Appendix A.8

**Table A7:** Comparison of endoscopic stricturotomy (ESt) with combined therapy (ESt + endoscopic ballon dilation).

ESt vs. Combined Therapy (ESt and EBD)					
		Lan, 2018 [12] *	Chaudhary, 2024 [23]		
		Combined Therapy	ESt	Combined Therapy	*p*-Value
	Follow-up	0.4 year (IQR 0.05–1.2)	NR	NR	
Patient’s characteristics	N	5	39/50	11/50	
	CD	5	NR	NR	
	Symptomatic	NR	NR	NR	
	Previous treatment	NR	NR	NR	
Stricture’s characteristics	Type	Anastomotic	Primary: 13/39 (33.3%) Anastomotic: 24/39 (61.5%)	Primary: 5/11 (45.5%) Anastomotic: 6/11 (54.5%)	*p* = 0.72
	Length	NR	1.2 ± 1 cm (SD)	2.76 ± 1.3 cm (SD)	*p* = 0.19
	Non-traversable	NR	NR	NR	
	Location		Ileocolonic: 5 (12.8%); Ileorectal: 1 (2.5%) Inlet and loop ileostomy: 1 (2.5%); Anal: 3 (7.6%); cecum: 2 (5.1%); Colonic: 14 (35.8%); Ileum: 7 (17.9%); Ileocecal anastomosis: 1 (2.5%); Rectum: 3 (7.6%); Recto-sigmoid colon: 2 (5.1%)	Ileorectal: 1 (9%); Anal: 1 (9%); Colonic: 4 (36.3%); Ileum: 1 (9%); Rectum: 4 (36.3%)	*p* = 0.39
Efficacy outcomes	Technical success	5 (100.0%)	NR	NR	
	Clinical success	1/2 (50.0%)	NR	NR	
	Endoscopic stricture improvement	1/2 (50.0%)	NR	NR	
Adverse events	Adverse Events	NR	4/39 (10.3%)	0/11 (0%)	*p* = 0.87
	Bleeding	0 (0.0%)	3/39 (7.7%)	0/11 (0%)	*p* = 0.74
	Perforation	0 (0.0%)	NR	NR	
	Additional Est/EBD	0 (0.0%)	15/39 (38.4%)	7/11 (63.6%)	*p* = 0.17
	Additional surgery	0 (0.0%)	1/39 (2.5%)	0/11 (0%)	*p* = 0.99
	Stricture-related ER visit	0 (0.0%)	NR	NR	
	ER-related hospitalization	0 (0.0%)	NR	NR	
	Escalation of medication	0 (0.0%)	NR	NR	

* Lan (2018) [12] compared only ESt versus EBD (Table A6) and reported outcomes from an additional group of five patients who underwent combined ESt + EBD; however, these patients were not included in the initial analysis, and no comparative analysis was performed.
